# The prevalence of underweight, overweight and obesity and their related socio-demographic and lifestyle factors among adult women in Myanmar, 2015-16

**DOI:** 10.1371/journal.pone.0194454

**Published:** 2018-03-16

**Authors:** Seo Ah Hong, Karl Peltzer, Kyi Tun Lwin, La Seng Aung

**Affiliations:** 1 ASEAN Institute for Health Development, Mahidol University, Nakhon Pathom, Thailand; 2 Institute for Health and Society, Hanyang University, Seoul, Republic of Korea; 3 Department for Management of Science and Technology Development, Ton Duc Thang University, Ho Chi Minh City, Vietnam; 4 Faculty of Pharmacy, Ton Duc Thang University, Ho Chi Minh City, Vietnam; Medical University of Vienna, AUSTRIA

## Abstract

**Background:**

The aim of the study was to estimate the prevalence of underweight and overweight or obesity and their socio-demographic and lifestyle factors in a female adult population in Myanmar.

**Material and methods:**

In a national cross-sectional population-based survey in the 2015–16 Myanmar Demographic and Health Survey, 12,160 women aged 18–49 years and not currently pregnant completed questionnaires and anthropometric measurements. Nutritional status was determined using Asian body mass index cut-offs: underweight (BMI<18.5 kg/m^2^), overweight (23.0–27.4 kg/m^2^), and obesity (≥27.5 kg/m^2^). Multinomial logistic regression modelling was used to determine the association between socio-demographic and lifestyle factors and weight status.

**Results:**

The prevalence of underweight was 14.1%, overweight 28.1% and obesity 13.1%. Among different age groups, the prevalence of underweight was the highest among 18 to 29 year-olds (20.2%), while overweight or obesity was the highest in the age group 30 to 49 years (around 50%). In multinomial logistic regression, being 30 to 49 years old, poorer and richer wealth status, living in all the other regions of Myanmar and ever contraceptive use were inversely and current tobacco use, not working and having less than two children ever born were positively associated with underweight relative to normal weight. Older age, having secondary education, urban residence, wealthier economic status, living with a partner, living in the Northern and Southern regions of Myanmar, having less than two children ever born and having ever used contraceptives were positively and current tobacco use was negatively associated with overweight or obesity relative to normal weight.

**Conclusions:**

A dual burden of both underweight and overweight or obesity among female adults was found in Myanmar. Sociodemographic and health risk behaviour factors were identified for underweight and overweight or obesity that can guide public health interventions to address both of these conditions.

## Introduction

The global prevalence of underweight decreased from 14.6% to 9.7%, and the prevalence of obesity increased from 6.4% to 14.9% among women from 1975 to 2014 [1]. In 2008, the prevalence of overweight (≥25 kg/m^2^) was 24% and obesity (≥30 kg/m^2^) 6% in Myanmar [2]. In a small study among university students in Yangon, Myanmar, 22.9% of female university students were overweight or obese (≥23 kg/m^2^)[3]. In countries of the Southeast Asian region, the prevalence of underweight and overweight or obesity were, in Bangladesh (35 years and older women), 36.0% underweight and 24.4% overweight or obesity (≥23 kg/m^2^)[4]; in Malaysia (18 years and above women) 52.9% overweight or obesity (≥25 kg/m^2^)[5], and in Vietnam among 25 to64 year old women in 2005, 21.9% underweight and 26.1% overweight or obesity (≥23 kg/m^2^) [6]. As globally, a decrease in the prevalence of underweight and increase of overweight or obesity have been reported in the Southeast Asian region, such as in Vietnam, over the past 20 years [6].

Undernutrition in adulthood can lead to increased morbidity and mortality and other adverse outcomes [7]. Obesity is a major risk factor for a number of non-communicable diseases, such as “diabetes mellitus, cardiovascular disease, hypertension and stroke, and certain forms of cancer” leading to increased morbidity and premature mortality [8]. Factors related to adult underweight may include socio-demographic variables, such as early adulthood (15–24 years) [9], having lower education [4,9–11], poorer economic background [4,9,11], not working [4], and residing in rural areas [6]. For example in India, among “rural young (15–24 years) females from more educated villages had a higher likelihood of underweight relative to those in less educated villages; but for rural mature (>24 years) females the opposite was the case” [11].

Socio-demographic risk factors for overweight or obesity may include being middle aged [4, 9], having higher education [4], higher economic status [4, 9, 11] and residing in urban areas [4, 6]. Hormonal contraception use has been found to increase the risk for obesity [12] and injected depot medroxyprogesterone acetate increased also weight [13]. In a study in Taiwan, compared with individuals who had never chewed betel nut, former and current betel nut chewers had a higher prevalence of obesity [14].

Nationally-representative data on the nutritional status of women of reproductive age are very limited in Southeast Asian countries. In particular, there is very little information available on Myanmar, also known as Burma, a lower middle-income country. As Myanmar has the first non-military president since the military coup of 1962 through general election in November 2015, Myanmar is expected to see a major shift. Myanmar is the largest country in mainland Southeast Asia, and its total population increased from 34.5 million in 1980 to 51.9 million in 2010 [15]. Myanmar people comprise of over 130 ethnic groups with 8 major groups [15]. Myanmar has a high prevalence of under-nutrition in children under five, which can influence adult weight status and non-communicable diseases that are estimated to account for 60% of total death [16]. There is a lack of more recent national data on the prevalence of underweight and overweight and obesity and its socio-demographic and behavioural factors in Myanmar. Therefore, it is important to understand factors driving the dual underweight and obesity burden in Myanmar so as to better design health interventions.

## Materials and methods

### Sample and procedure

This study is a secondary data analysis of the 2015–16 Myanmar Demographic and Health Survey (MDHS), which is a cross-sectional nationally representative population-based survey. The MDHS is the first MDHS and was conducted by the Ministry of Health and Sports (MoHS) [17]. The MDHS utilized a two-stage (442 clusters or enumeration areas and 30 households per cluster) sample of households, stratified by urban and rural areas in 15 states and regions [17]. A more detailed description of the survey procedure has been published elsewhere [17]. A total of 12,885 women aged 15 to 49 years participated in MDHS (response rate 96%) [17]. The final sample for analyses included 11,078 women, excluding 510 who were currently pregnant, 215 who had no Body Mass Index (BMI) and 1,082 who were under 18 years of age. The datasets of the MDHS are free to download and were accessed from the DHS website after permission to use the MDHS data in this analysis was obtained from Opinion Research Corporation (ORC) Macro Inc.

### Measures

#### Socio-demographic and life style variables

Socio-demographic variables including age, formal education, living arrangement, number of living children, residence, wealth status, and husband’s or partner’s education were collected by questionnaire that was administered [17]. A household wealth index was calculated using the household's ownership of selected assets (e.g., bicycle or car, source of drinking water) [17]. Life style variables included ever contraceptive use, current tobacco use and chewing betel nuts.

#### Anthropometric measurements

Weight and height were measured “using measuring boards specially made by Shorr Productions for use in survey settings and lightweight SECA scales with digital screens at the participant's home by trained field research staff” [17]. BMI was calculated as weight (kg)/height (m^2^). Asian specific BMI cut-offs were used to define underweight (<18.5 kg/m^2^), overweight (23.0 to <27.5 kg/m^2^) and obese (≥27.5 kg/m^2^) [18].

### Data analysis

Descriptive statistics were used to present the unweighted number and weighted proportion of general subject characteristics and outcome variables. Chi-square tests were used to identify differences in proportions of the categories of the exposure by nutritional status of women. To determine associations between socio-demographic factors and nutritional status multinomial logistic regression tests were used. Dependent variables were underweight and overweight or obesity and the comparison group was women with normal weight. Odds ratios (ORs) and 95% confidence intervals (CIs) after adjustment for covariates were estimated and presented. All analyses conducted took the sampling design parameters, weighting, clustering, and stratification of the study survey into account. All statistical analyses were done in SAS 9.4 (SAS Institute, Cary, NC).

## Results

### Sample characteristics

The total sample in the current analyses included 11,078 women (age range of 18–49 years) in Myanmar. The proportion of women who had secondary or more education was 46%, 59% were living with a partner, the majority (67.5%) were working, and living in rural areas (71%). About half of the women (49%) had ever used contraceptives and 18% and 3.8% had used betel nuts and tobacco, respectively (Table 1).

**Table 1:** General characteristics of women (n = 11,078) aged 18–49 years old participating in 2015–16 Myanmar Demographic Health Survey.

		Unweighted number	(%)
Current age of mothers			
	18–29	4120	(36.8)
	30–39	3656	(33.8)
	40–49	3302	(29.4)
Education			
	No education	1493	(12.4)
	Primary school	4846	(41.3)
	Secondary	4569	(36.1)
	College +	1250	(10.2)
Working status			
	No	4274	(32.5)
	Yes	7881	(67.5)
Marital status			
	Living with partner	7259	(59.0)
	Living without partner	4901	(41.0)
Wealth index			
	Poorest	2182	(17.2)
	Poorer	2314	(18.8)
	Middle	2520	(20.8)
	Richer	2593	(21.0)
	Richest	2551	(22.2)
Place of residence			
	Urban	3566	(29.0)
	Rural	8594	(71.0)
Geographical area			
	North	734	(2.8)
	Northwest	1702	(12.0)
	West	830	(5.8)
	Southwest	1868	(27.6)
	South	1436	(5.8)
	East	1417	(10.6)
	South east	698	(2.3)
	Central	3475	(33.0)
Total number of children ever born			
	<2	10589	(89.4)
	2+	1571	(10.6)
Ever contraceptive use			
	No	6298	(50.6)
	Yes	5862	(49.4)
Chew betel nuts (yes)			
	No	9650	(81.9)
	Yes	2510	(18.1)
Current tobacco use ^1^^)^			
	No	11532	(96.2)
	Yes	628	(3.8)

All values are presented as unweighted number and weighted percentages

^1)^ Including smoking cigarettes/pipe/cheroot, chewing tobacco, snuff or other forms of tobacco

The prevalence of underweight was 14.1% and overweight or obesity 41.1% (overweight 28.1% and obesity 13.1%) (Table 2). Among different age groups, underweight was the highest among 18–29 year-olds (20.2%), while overweight or obesity was the highest in the age group 30 to 49 years (around 50%). Women who had college or higher education, came from rich a household, lived with a spouse,had less than two children, resided in urban areas, ever used contraception, currently used tobacco and chew betel nuts had a higher prevalence of overweight or obesity.

**Table 2:** Nutritional status by general characteristics of women aged 15–49 years old participating in 2015–16 Myanmar Demographic Health Survey (n = 11,078).

		Prevalence of weight status (%)								P-value
		Underweight (<18.5)		Normal weight (18.5–22.9)		Overweight (23.0–27.4)		Obese (≥27.5)		
		n	(%)	n	(%)	n	(%)	n	(%)	
Total prevalence		1477	(14.1)	5090	(44.8)	3105	(28.1)	1406	(13.1)	
Current age of mothers										
	18–29	773	(20.2)	2,293	(53.7)	820	(20.0)	234	(6.1)	< .0001
	30–39	371	(10.2)	1,565	(42.4)	1,165	(31.9)	555	(15.5)	
	40–49	333	(10.9)	1,232	(36.4)	1,120	(33.7)	617	(19.0)	
Education										
	No education	206	(14.8)	744	(50.0)	370	(25.6)	121	(9.6)	0.0004
	Primary school	561	(13.0)	2,128	(45.4)	1,315	(28.5)	616	(13.0)	
	Secondary	530	(15.4)	1,682	(42.6)	1,083	(28.4)	487	(13.6)	
	College +	180	(13.6)	535	(42.8)	337	(28.1)	181	(15.5)	
Working status (yes)		987	(13.9)	3,408	(45.6)	2,066	(27.7)	931	(12.8)	0.2374
Marital status										
	Living with partner	736	(10.9)	3,095	(41.4)	2,262	(31.8)	1,110	(15.9)	< .0001
	Living without partner	741	(19.8)	1,995	(50.9)	843	(21.3)	296	(8.0)	
Wealth index										
	Poorest	363	(19.8)	1,105	(53.4)	393	(20.0)	123	(6.8)	< .0001
	Poorer	281	(14.5)	1,080	(50.4)	549	(26.3)	187	(8.8)	
	Middle	301	(14.5)	1,040	(44.3)	693	(30.0)	250	(11.2)	
	Richer	275	(12.3)	1,004	(42.5)	715	(29.7)	372	(15.6)	
	Richest	257	(10.8)	861	(36.1)	755	(32.3)	474	(20.8)	
Place of residence										
	Urban	363	(11.0)	1,229	(36.7)	1,050	(32.8)	592	(19.5)	< .0001
	Rural	1,114	(15.3)	3,861	(48.0)	2,055	(26.2)	814	(10.5)	
Geographical area										
	North	49	(9.6)	273	(42.6)	225	(31.1)	118	(16.7)	< .0001
	Northwest	163	(11.6)	789	(45.4)	435	(29.5)	164	(13.5)	
	West	139	(18.5)	408	(54.2)	156	(21.0)	48	(6.3)	
	Southwest	236	(13.8)	679	(40.3)	517	(30.5)	267	(15.4)	
	South	173	(13.1)	549	(42.6)	372	(28.2)	207	(16.1)	
	East	95	(7.0)	666	(50.5)	352	(27.3)	160	(15.2)	
	South east	76	(12.3)	274	(43.8)	188	(29.1)	94	(14.8)	
	Central	546	(17.4)	1,452	(45.5)	860	(26.7)	348	(10.4)	
Total number of children ever born										
	<2	1,318	(14.3)	4,277	(43.9)	2,726	(28.3)	1,281	(13.5)	< .0001
	2+	159	(12.3)	813	(51.9)	379	(26.4)	125	(9.4)	
Ever contraceptive use										
	No	924	(18.7)	2,667	(49.5)	1,204	(22.5)	463	(9.3)	< .0001
	Yes	553	(10.1)	2,423	(40.8)	1,901	(32.8)	943	(16.3)	
Chew betel nuts										
	No	1,165	(14.3)	3,963	(45.1)	2,414	(27.9)	1,081	(12.7)	0.2729
	Yes	312	(13.4)	1,127	(43.5)	691	(28.6)	325	(14.5)	
Tobacco use ^1^^)^										
	No	1,358	(13.6)	4,769	(44.6)	2,967	(28.4)	1,362	(13.3)	< .0001
	Yes	119	(25.4)	321	(48.5)	138	(19.3)	44	(6.9)	

All values are presented as unweighted number and weighted percentages

^1)^ Including smoking cigarettes/pipe/cheroot, chewing tobacco, snuff or other forms of tobacco

**Fig 1:**
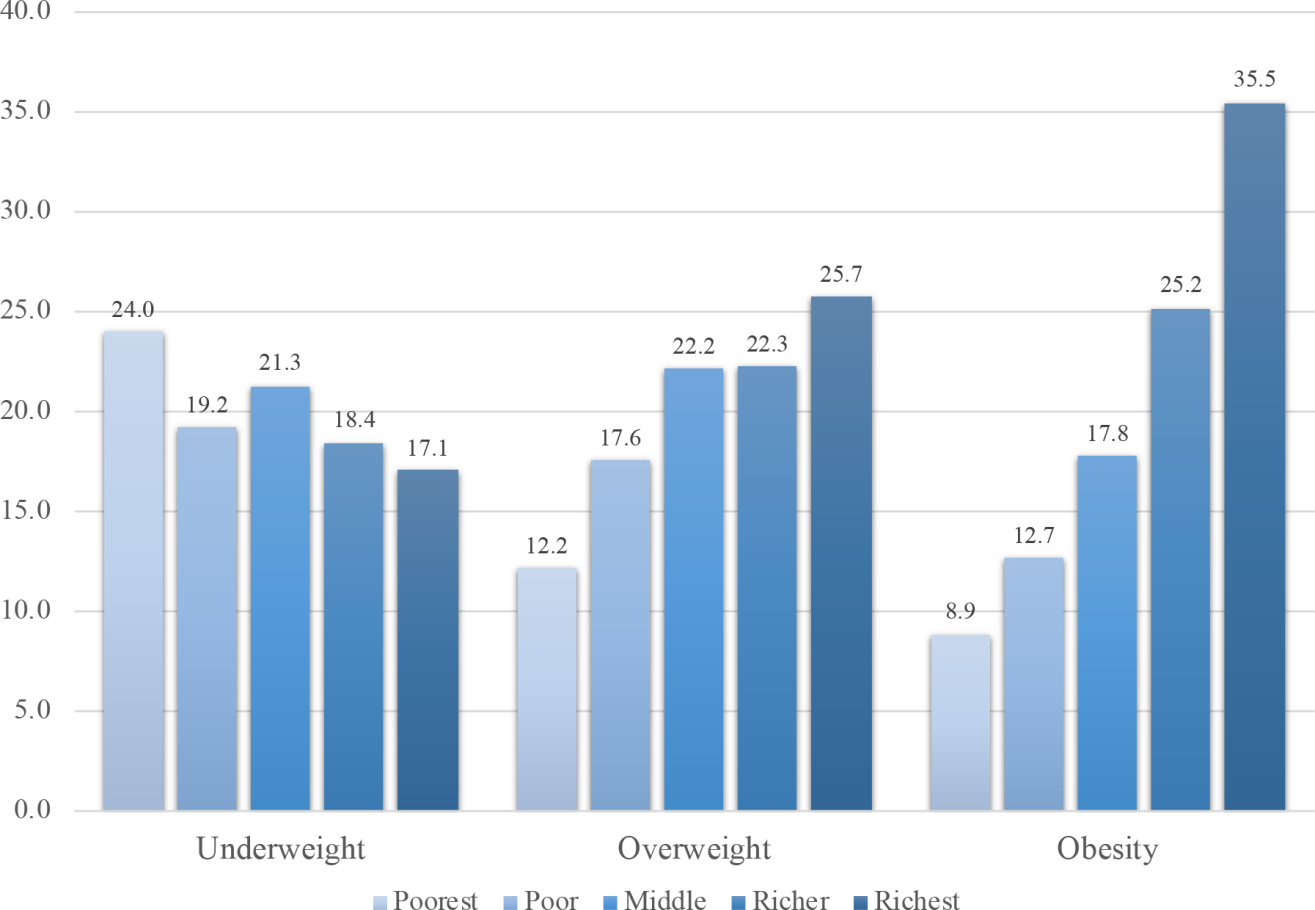
Distribution of household wealth index quintiles by nutritional status for Myanmar women.

**Fig 2:**
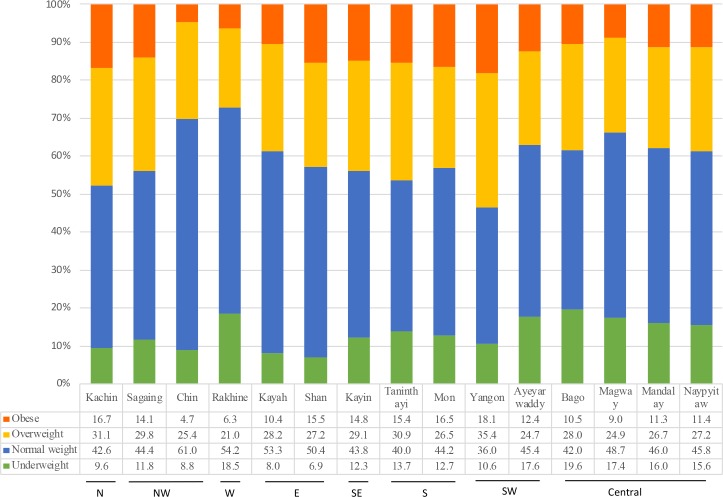
Prevalence of underweight, overweight, and obesity of women living in the 15 states and regions of Myanmar.

Prevalence of underweight, overweight and obesity differed by wealth quintiles and study regions, as shown in Figs 1 and 2. The prevalence of underweight by wealth quintiles ranged from 24.0% in the poorest to 17.1% in the richest households (Fig 1). Wealth disparity was grater in the prevalence of overweight or obesity than in the prevalence of underweight: 12.2% in the poorest to 25.7% in the richest households for overweight, and 8.9% to 35.5%, respectively, for obesity. In addition, the prevalence of nutritional status seemed to differ by the 15 regions or states (Fig 2). The prevalence of underweight ranged from 7%-9% in Chin, Shan, and Kayah to 18.5% in Rakhine and 19.6% in Bago. The prevalence of overweight or obesity ranged from 27.3% in Rakhine to 53.5% in Yangon. Northern and southern Myanmar had a higher prevalence of obesity than other regions in Myanmar.

### Associations with underweight and overweight or obesity

Table 3 shows the ORs for underweight and overweight or obesity relative to normal weight for the covariates considered in the analysis. Compared to 18 to 29 year old women, older women were less likely to be underweight and were more likely to be overweight or obese, relative to normal weight. Women with secondary education were more likely to be overweight or obese (OR = 1.22 95% CI = 1.01–1.48), and women who were not working were more likely to be underweight (OR = 1.17, 95% CI = 1.00–1.36). Persons with a richer economic status (OR = 2.61, 95% CI = 2.10–3.24), living with a partner (OR = 1.57, 95% CI = 1.35–1.83) and residing in an urban area (OR = 1.41, 95% CI = 1.19–1.66) were positively associated with overweight or obesity. The odds of being underweight were lower among participants who had ever used contraceptives (OR = 0.74, 95% CI = 0.61–0.89), and were higher in those who had less than two children (OR = 1.28, 95% CI = 1.02–1.60) and who currently used tobacco (OR = 2.01, 95% CI = 1.50–2.69). The odds of being overweight or obese were higher among women who had less than two children (OR = 1.20, 95% CI = 1.02–1.40) and who had ever used contraceptives (OR = 1.43, 95% CI = 1.25–1.64) and were lower among those who currently used tobacco (OR = 0.57, 95% CI = 0.44–0.75). Compared to living in the central region of Myanmar, the odds of being underweight were lower in most of the other areas, and the odds of being overweight or obese were higher in the northern, southern, southwestern and southeastern regions in Myanmar (Table 3).

**Table 3:** Odds ratios (OR) for factors associated with underweight and overweight or obesity relative to normal weight.

		Underweight(<18.5) vs. Normal weight (18.0–22.9)			Overweight (≥23) vs. Normal weight (18.0–22.9)		
		OR	(95% CI)		OR	(95% CI)	
Current age of mothers							
	18–29	1.00			1.00		
	30–39	**0.68**	**(0.56**	**0.82)**	**2.07**	**(1.81**	**2.37)**
	40–49	**0.76**	**(0.64**	**0.92)**	**2.74**	**(2.40**	**3.13)**
Education							
	No education	1.00			1.00		
	Primary school	0.86	(0.68	1.09)	1.17	(0.99	1.37)
	Secondary	1.06	(0.82	1.38)	**1.22**	**(1.01**	**1.48)**
	College +	0.96	(0.71	1.29)	1.00	(0.78	1.28)
Working status							
	No	**1.17**	**(1.00**	**1.36)**	1.02	(0.91	1.15)
	Yes	1.00			1.00		
Marital status							
	Living with partner	0.88	(0.72	1.06)	**1.57**	**(1.35**	**1.83)**
	Living without partner	1.00			1.00		
Wealth index							
	Poorest	1.00			1.00		
	Poorer	**0.77**	**(0.62**	**0.95)**	**1.34**	**(1.14**	**1.59)**
	Middle	0.80	(0.63	1.02)	**1.84**	**(1.53**	**2.22)**
	Richer	**0.72**	**(0.56**	**0.91)**	**2.01**	**(1.66**	**2.43)**
	Richest	**0.69**	**(0.53**	**0.91)**	**2.61**	**(2.10**	**3.24)**
Place of residence							
	Urban	0.95	(0.78	1.15)	**1.41**	**(1.19**	**1.66)**
	Rural	1.00			1.00		
Geographical area							
	North	0.56	(0.31	1.01)	**1.41**	**(1.12**	**1.77)**
	Northwest	**0.64**	**(0.51**	**0.81)**	1.23	(0.98	1.55)
	West	**0.68**	**(0.51**	**0.91)**	0.94	(0.71	1.23)
	Southwest	0.84	(0.68	1.03)	**1.35**	**(1.12**	**1.63)**
	South	**0.73**	**(0.57**	**0.94)**	**1.33**	**(1.08**	**1.65)**
	East	**0.34**	**(0.23**	**0.52)**	1.16	(0.94	1.43)
	Southeast	**0.59**	**(0.46**	**0.77)**	**1.34**	**(1.09**	**1.65)**
	Central	1.00			1.00		
Total number of children ever born							
	<2	**1.28**	**(1.02**	**1.60)**	**1.20**	**(1.02**	**1.40)**
	2+	1.00			1.00		
Ever contraceptive use							
	No	1.00			1.00		
	Yes	**0.74**	**(0.61**	**0.89)**	**1.43**	**(1.25**	**1.64)**
Chew betel nuts							
	No	1.00			1.00		
	Yes	0.97	(0.79	1.21)	1.12	(0.97	1.29)
Tobacco use ^1^^)^							
	No	1.00			1.00		
	Yes	**2.01**	**(1.50**	**2.69)**	**0.57**	**(0.44**	**0.75)**

Nutritional status was classified as underweight (BMI <18.5 Kg/m^2^), normal weight (18.0–22.9 kg/m^2^), overweight or obesity (≥23 Kg/m^2^)

^1)^ Including smoking cigarettes/pipe/cheroot, chewing tobacco, snuff or other forms of tobacco

## Discussion

The results of this national study demonstrate the co-existence of a dual burden of underweight (14.1% for BMI <18.5 kg/m^2^) and overweight/obesity (41.1% for BMI ≥23 kg/m^2^) among 18 years and older women in 2015–16 in Myanmar. These figures seem to to roughly comparable with older previous studies in Myanmar [2, 3]. The prevalence of underweight found in this study was lower than in Bangladesh and Vietnam [4, 6], and the prevalence of overweight or obesity was lower than in Malaysia [5] but higher than in 2008 in Vietnam [6].

The study found that the prevalence of underweight was the highest among young adults (18–29 years). This finding could be linked to an association of not working status and having a smaller number of children with underweight in this study, as it may be partly explained that young people are more likely to have fewer children as they are studying or delaying marriage. Reasons for the high prevalence of underweight during early adulthood may be related to food insecurity [19] and fear of being fat [20]. Some studies report an increase of an underweight body ideal and in eating disorders in Southeast Asia [21]. In a small study among university students in Myanmar 20.2% of female university students reported disordered eating attitudes [22].

In contrast to previous studies [4, 6, 9–11], this study did not find an association between educational levels, place of residence and underweight. Meanwhile, this study found an association between poorer economic background and underweight, which is in agreement with some previous studies [4,9,11,19]. The study found that compared to living in the central region of Myanmar, the odds of being underweight relative to normal weight were lower in all the other regions. This may be because as one of the most prevalent areas of underweight in Myanmar is the central area, including Bago, Magway, Mandalay, and Nay Pyi Taw (19.6%, 17.4%, 16.0%, and 15.6%, respectively as shown in Fig 2). The population of Myanmar is most heavily concentrated in the central part of the country, along a corridor connecting the cities of Yangon, Nay Pyi Taw and Mandalay, as approximately 50 per cent of the total population lives within 100 kilometres of these three urban centres [23]. It is assumed that the rapid growth of cities together with the growth of the urban poor made health inequities worse within cities, which may lead to higher prevalence of underweight in the urban poor [24]. In addition, about 80% of the population in the Bago region, being the second largest rice producer of all states/regions in the country [25], relies on agriculture for their livelihoods [26]. Although no association of place of residence with underweight was found in this study, the rural population may have a generally poorer nutritional status than the urban population. Contraceptive use was in this study found to have an inverse association with underweight relative to normal weight. This finding may be supported by a study among women in Nigeria that also found an association between non-contraceptive use and underweight [27]. Finally, current tobacco users were more likely to be underweight, as found in previous studies [28–30].

Regarding overweight or obesity, in consistence with previous studies [4, 6, 9, 11], this study found that being middle aged, having secondary education, higher economic status and urban residence were associated with overweight or obesity. With regard to age, the positive linear association could potentially be due to pregnancy and weight retention associated with child birth and the metabolic slow-down associated with age. This is also supported by our results that women with less than two children ever born were more likely to be overweight as well as underweight relative to normal weight. Generally, obesity is associated with lower socioeconomic status in developed countries [31], while it is more often profound in privileged households in lower income countries as shown in our study. This suggests better food availability and a sedentary lifestyle in a more food-abundant environment of advantaged households can be associated with overweight and/or obesity in Myanmar. Further, the study found that compared to living in the central region of Myanmar, the odds of being overweight or obese were higher in the northern and southern (including southwestern and southeastern) regions in Myanmar.

This result supports the specificity of food and lifestyle practices in each region in Myanmar, implying the importance of identifying the differing risk factors leading to obesity in the different regions. In the northern area of Myanmar, Kachin, which is located close to the China border, the majority of the people have an eating pattern like in Chinese culture. Although they consume little oil, their eating pattern consists mainly of rice, noodle and lot of meat like Chinese people. Meanwhile, in the southern area (southeast, southwest and south), they produce large amounts of agricultural products, such as rice and vegetables due to enough water supply. In addition, oil consumption is increasing compare to past years, as the government allowed to import oil from Thailand and other countries, which is cheap and easily available. This may promote the habit of eating much rice and much oil. Another factor may be the impact of urbanization in these areas, since they serve ascommercial, political, and administrative hub areas in Myanmar. In addition, urbanization may contribute to physical inactivity among women in the areas. As previously found [12,13,27], this study identified that contraceptive use increased the risk for overweight or obesity. This may be linked to contraceptive users having better education and coming from higher income households resulting in greater exposure to information from the media [27,32]. Our study showed current tobacco users were less likely to be overweight or obese compared to having normal weight, in accordance with other studies [29], although whether smokers have more abdominal obesity is controversial as some studies reported current smokers had more abdominal obesity than never smokers [30]. For betel nut chewing behavior, this study did not find an association with overweight or obesity. Several studies in Taiwanese men showed a positive dose-response association between betel nut consumption and general and central obesity [14,33], probably by increasing appetite [33]. Chewing betel nut is extremely popular in Myanmar, like other Southeast Asian countries. It may lead to a need for further studies in Myanmar.

### Study limitations

The study was a cross-sectional study and the temporal relationships between socio-demographic factors and health risk behaviours and underweight and overweight or obesity cannot be established in such studies; further longitudinal studies are needed. Apart from anthropometric measurements, a limitation of the study was that all the other information was collected based on self-reporting.

## Conclusion

The study found a high prevalence of both underweight and overweight or obesity among 18 to 49 year-old women in 2015–16 in Myanmar. Sociodemographic and health behaviour risk factors of underweight and overweight or obesity were identified which can guide much needed public health interventions to address both these conditions. This implies that as both conditions are associated with an increased risk of developing non-communicable diseases, public health interventions to address both conditions and associated risk factors should be promoted to improve the health of the Myanmar women.

## Abbreviations

BMI: Body Mass Index
MDHS: Myanmar Demographic and Health Survey
MoHS: Ministry of Health and Sports (MoHS)
